# Enteral Nutrition in Pediatric Patients Undergoing Hematopoietic SCT Promotes the Recovery of Gut Microbiome Homeostasis

**DOI:** 10.3390/nu11122958

**Published:** 2019-12-04

**Authors:** Federica D’Amico, Elena Biagi, Simone Rampelli, Jessica Fiori, Daniele Zama, Matteo Soverini, Monica Barone, Davide Leardini, Edoardo Muratore, Arcangelo Prete, Roberto Gotti, Andrea Pession, Riccardo Masetti, Patrizia Brigidi, Silvia Turroni, Marco Candela

**Affiliations:** 1 Microbial Ecology of Health Unit, Department of Pharmacy and Biotechnology, University of Bologna, Via Belmeloro 6, 40126 Bologna, Italy; federica.damico8@unibo.it (F.D.); elena.biagi@unibo.it (E.B.); simone.rampelli@unibo.it (S.R.); matteo.soverini5@unibo.it (M.S.); monica.barone@unibo.it (M.B.); patrizia.brigidi@unibo.it (P.B.); silvia.turroni@unibo.it (S.T.); 2 Department of Chemistry, University of Bologna, Via Selmi 2, 40126 Bologna, Italy; jessica.fiori@unibo.it; 3 Pediatric Oncology and Hematology Unit “Lalla Seràgnoli”, Department of Pediatrics, University of Bologna, Sant’Orsola Malpighi Hospital, Via Massarenti 9, 40138 Bologna, Italy; daniele.zama@gmail.com (D.Z.); davide.leardini3@gmail.com (D.L.); edoardo.muratore@gmail.com (E.M.); arcangelo.prete@unibo.it (A.P.); andrea.pession@unibo.it (A.P.); riccardo.masetti@gmail.com (R.M.); 4 Department of Pharmacy and Biotechnology, University of Bologna, Via Belmeloro 6, 40126 Bologna, Italy; roberto.gotti@unibo.it

**Keywords:** enteral nutrition, parenteral nutrition, gut microbiota, short-chain fatty acids, hematopoietic stem cell transplantation, graft-versus-host disease

## Abstract

Hematopoietic stem cell transplantation (HSCT) is the first-line immunotherapy to treat several hematologic disorders, although it can be associated with many complications reducing the survival rate, such as acute graft-versus-host disease (aGvHD) and infections. Given the fundamental role of the gut microbiome (GM) for host health, it is not surprising that a suboptimal path of GM recovery following HSCT may compromise immune homeostasis and/or increase the risk of opportunistic infections, with an ultimate impact in terms of aGvHD onset. Traditionally, the first nutritional approach in post-HSCT patients is parenteral nutrition (PN), which is associated with several clinical adverse effects, supporting enteral nutrition (EN) as a preferential alternative. The aim of the study was to evaluate the impact of EN vs. PN on the trajectory of compositional and functional GM recovery in pediatric patients undergoing HSCT. The GM structure and short-chain fatty acid (SCFA) production profiles were analyzed longitudinally in twenty pediatric patients receiving HSCT—of which, ten were fed post-transplant with EN and ten with total PN. According to our findings, we observed the prompt recovery of a structural and functional eubiotic GM layout post-HSCT only in EN subjects, thus possibly reducing the risk of systemic infections and GvHD onset.

## 1. Introduction

Hematopoietic stem cell transplantation (HSCT) is a clinical practice routinely used to treat patients with high-risk hematopoietic malignancies and hematological and genetic diseases. The HSCT procedure includes a conditioning regimen, treatments with chemotherapy and/or immunotherapy, which aim to eliminate cancer cells while allowing patients to receive donor HSCs [1,2]. One of the most life-threating complications of these transplant procedures, which can lead to patient morbidity and mortality, is acute graft-versus-host disease (aGvHD), characterized by the response of alloreactive donor T cells to host organs including skin, gut, liver, lungs and central nervous system [1,2,3]. The gut microbiome (GM) has been hypothesized to have a role in aGvHD onset [4,5,6]. In particular, several studies have been realized in this field, indicating that the path of GM recovery following HSCT is closely related to the risk of developing aGvHD [4,7]. Indeed, HSCT conditioning regimens, in addition to damaging the intestinal epithelial barrier, induce the disruption of the GM structure, with the loss of all its probiotic properties. A prolonged GM dysbiosis following HSCT has in turn been demonstrated to expose the patient to an increased risk of innate immune system activation (i.e., alloreactive T cells) and systemic infections, causing aGvHD development [8,9]. Conversely, the prompt recovery of a eubiotic GM layout may provide the host with health-promoting GM-dependent ecological services protective against the aGvHD onset, such as barrier effect and preservation of immune homeostasis [10]. These GM-dependent probiotic activities mainly rely on a balanced production of short-chain fatty acids (SCFAs), i.e., the end-products of GM fermentation of complex non-digestible plant polysaccharides (e.g., cellulose, resistant starch, xylans and inulin) [11,12,13]. Indeed, SCFAs, mainly acetate, propionate and butyrate, have well-known health-promoting activities, which are important to mitigate the aGvHD development risk [14], being potent anti-inflammatory and antimicrobial compounds, as well as strengthening the epithelial barrier and regulating the host metabolic homeostasis [12,13,14,15]. Patients who undergo HSCT are often suffering from nutritional deficiencies, with a decrease in oral food intake, weight loss and malnutrition due to treatment side effects (e.g., nausea, vomiting, diarrhea and oral mucositis) [16,17]. Therefore, nutritional support after the transplant, by enteral nutrition (EN) or parenteral nutrition (PN), has become a strategic aspect to be considered in HSCT-associated procedures. EN, also called “tube-feeding”, is the method by which food is directly delivered inside the patient’s gastrointestinal tract, while PN is an effective strategy of delivering nutrients directly into the bloodstream. Nowadays, it is well known that total PN, the first-line approach for HSCT patients, is associated with different clinical adverse effects such as infections [18,19,20] and metabolic disorders [21], as well as with gut mucosal atrophy [22], cell dysfunction [23], and alterations in GM composition [24]. Conversely, EN has been indicated as a possible solution to overcome all these adverse effects. Further, we believe that EN, by feeding the GM, besides providing nutritional benefit, can also favor the prompt recovery of a eubiotic and SCFA-producing GM layout, with ultimate benefits in terms of aGvHD protection. For all the reasons that link GM composition and its metabolites to post-HSCT outcomes, as well as in light of the importance of nutritional intake to maintain an “appropriate” GM–host relationship, here, we evaluated the efficacy of EN, in comparison with the traditional PN, in supporting the more rapid recovery of a eubiotic GM layout in pediatric patients undergoing HSCT. In particular, we analyzed longitudinal fecal samples from twenty pediatric patients to build GM and SCFA production trajectories from before to up to 120 days after the transplant; ten of them were fed with EN post-HSCT, while the rest were fed with PN.

## 2. Materials and Methods

### 2.1. Subject Enrollment and Sample Collection

**Figure 1:**
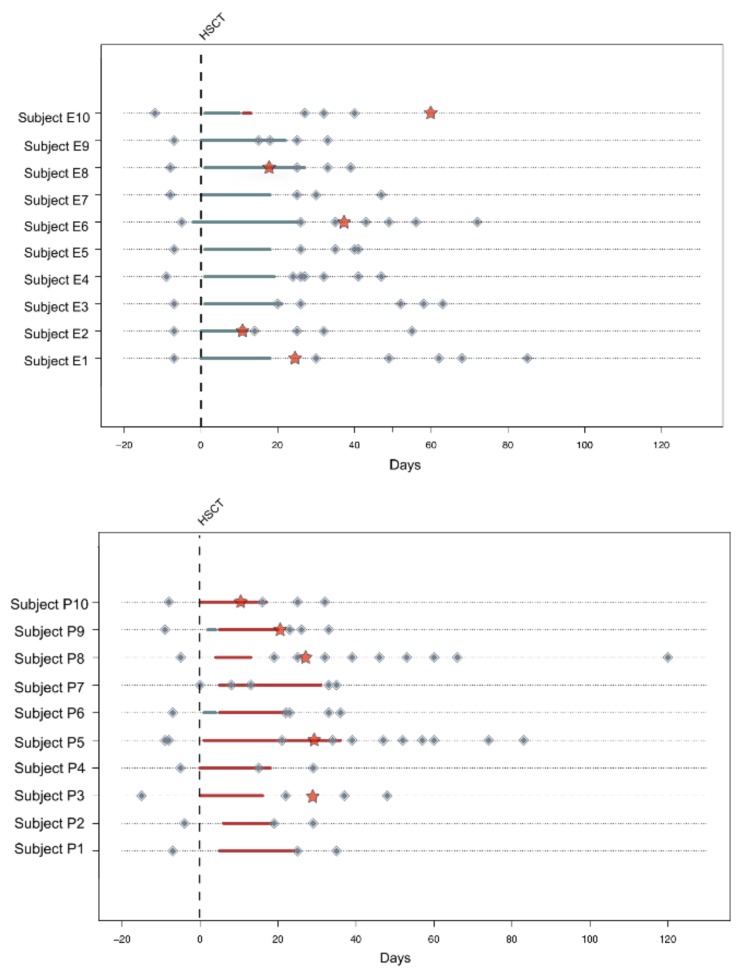
General representation of the fecal sampling for each enrolled subject. On the top of the figure are depicted the ten patients that followed EN (enteral nutrition), while at the bottom are highlighted the ten subjects treated with PN (parenteral nutrition). Diamonds indicate each individual fecal sample taken pre and post-transplant. Hematopoietic stem cell transplantation (HSCT) is indicated by the vertical line in the graph and the moment of the eventual acute graft-versus-host disease (aGvHD) occurrence is reported with a red star. The bars below each subject’s timeline indicate the type and the length of the nutritional regimen followed: blue bars indicate EN and red bars indicate PN.

A total of twenty patients who underwent HSCT were enrolled for a longitudinal study on fecal samples approved by the Ethics Committee of the Sant’Orsola-Malpighi Hospital-University of Bologna (ref. number 19/2013/U/Tess). Written informed consent was obtained from each enrolled patient or parent/legal guardian. Study inclusion parameters were the availability of a pre-HSCT fecal sample and of at least two samples collected after HSCT. Patients were clustered in two groups depending on the feeding procedure used the days immediately after the transplant—ten patients were fed by using EN (mean age 9.3 years) and ten patients were treated with the administration of total PN (mean age 10.1 years). Information regarding the enteral mixture administered to the patients is represented in Supplementary Table S1. Inclusion criteria for the nutritional regimen clustered children that received EN nutrition for more than 7 days post-HSCT in EN group and all patients who received total PN or EN feeding for less than 7 days after the transplant in PN group. Patients in the PN group were chosen based on matching the ones in the EN group in terms of age, source of stem cell, type of disease and conditioning regimen. Before the transplant, all patients received trimethoprim-cotrimoxazole once a week for the prevention of *Pneumocystis jirovecii* infection and anti-fungal prophylaxis was also performed by using voriconazole or posaconazole. None of these patients were treated with antibiotic prophylaxis or gut decontamination. For both nutritional groups, five out of ten patients developed acute graft-versus-host disease (aGvHD) at different grades of severity (Supplementary Table S2). In all patients, febrile neutropenia occurred, and ceftazidime was used in all of them as the first-line antibiotic therapy. A total of 104 fecal samples were collected before HSCT and at different time points after the transplant, up to 120 days post-HSCT (Figure 1). Samples were stored at −20 °C and shipped in dry ice to the laboratory where the microbiological analysis was performed.

### 2.2. Microbial DNA Extraction

Total microbial DNA was extracted from 250 mg of stool sample using the repeated bead-beating plus column method, as previously described with few modifications [25]. Briefly, all the samples were suspended in 1 mL of lysis buffer (500 mM NaCl, 50 mM Tris-HCl, pH 8, 50 mM EDTA, and 4% sodium dodecyl sulfate) and bead-beaten three times in a FastPrep instrument (MP Biomedicals, Irvine, CA, USA) at 5.5 movements/s for 1 min, in the presence of four 3 mm glass beads and 0.5 g of 0.1 mm zirconia beads (BioSpec Products, Bartlesville, OK, USA). After an incubation step at 95 °C for 15 min, samples were centrifuged at 13,000 rpm for 5 min. Two hundred and sixty microliters of 10 M ammonium acetate was added to the supernatant, followed by a 5 min incubation in ice and a 10 min centrifugation at 13,000 rpm. Each sample was incubated with one volume of isopropanol, followed by an incubation in ice for 30 min. Precipitated nucleic acids were washed with 70% ethanol, re-suspended in 100 μL of TE (10 mM Tris-HCl, 1 mM EDTA pH 8.0) buffer, and treated with 2 μL of 10 mg/mL DNase-free RNase at 37 °C for 15 min. DNA purification was performed using the DNeasy Blood and Tissue Kit (QIAGEN, Hilden, Germany) following the manufacturer’s instructions. DNA concentration and quality were evaluated using NanoDrop ND-1000 spectrophotometer (NanoDrop Technologies, Wilmington, DE, USA).

### 2.3. 16S rRNA Gene Amplification and Sequencing

The V3–V4 hypervariable region of the 16S rRNA gene was amplified by using the 341F and 785R primers with linked Illumina adapter overhang sequences, as previously described in Klindworth et al. [26]. Fragment amplification was performed by using KAPA HiFi HotStart ReadyMix (Roche, Basel, CH, USA), setting the thermal cycle as follows: 3 min at 95 °C, 25 cycles of 30 s at 95 °C, 30 s at 55 °C, and 30 s at 72 °C, and a final 5 min step at 72 °C. Library preparation followed a first purification with a magnetic bead-based clean-up system (Agencourt AMPure XP, Beckman Coulter, Brea, CA, USA) and a limited-cycle PCR was performed to obtain the indexed library using Nextera technology, followed by a second AMPure XP magnetic beads clean-up step. Final libraries were prepared by pooling all samples to an equimolar concentration of 4nM; the denaturation and dilution to 5 pmol was carried out before performing the sequencing on an Illumina MiSeq platform with a 2 × 250 bp paired-end protocol according to manufacturer’s instructions (Illumina, San Diego, CA, USA). Raw sequence reads were deposited in the National Center for Biotechnology Information Sequence Read Archive (https://www.ncbi.nlm.nih.gov/bioproject/PRJNA592853).

### 2.4. Gas Chromatography-Mass Spectrometry Determination of Short-Chain Fatty Acids in Fecal Samples

When the amount of stool material was enough, an aliquot of each sample was weighted (approximately 250 mg), for a total of 99 fecal samples, and analyzed for SCFA determination. The analysis of acetic acid, propionic acid and butyric acid was performed as reported in several publications [27,28]. Briefly, samples were homogenized in 10% perchloric acid and centrifuged at 15,000 rpm for 5 min at 4 °C. Supernatants were diluted 1:10 in water and added with D8-butyric acid (internal standard) to 20 μg/mL. Headspace solid-phase micro extraction (HS-SPME) was carried out by using a 75 μm CarboxenTM/polydimethylsiloxane fiber (Supelco, Sigma-Aldrich, Milan, Italy) at 70 °C, with a 10 min equilibration and 30 min of extraction time. Analytes were desorbed into the gas chromatography (GC) injector and GC–mass spectrometry analysis was carried out on a TRACE GC 2000 Series (Thermo Fisher Scientific, Waltham, MA, USA) gas chromatograph, interfaced with GCQ Plus (Thermo Fisher Scientific, Waltham, MA, USA) mass detector with ion-trap analyzer. Phenomenex ZB-WAX (30 m × 0.25 mm ID, 0.15 μm film thickness) capillary column was used. SCFA concentration was expressed as micromoles per gram (μmol/g) of feces. Limit of detection ranged from 4 to 68 nmol/g.

### 2.5. Bioinformatics and Statistical Analysis

A mean of 8043.9 ± 3632.5 (mean ± SD) high-quality sequences per sample was obtained. Raw sequences were processed using a pipeline that combined PANDASeq [29] and QIIME2 [30]. After length (minimum/maximum = 250/550 bp) and quality filtering (default parameters), reads were cleaned and binned into amplicon sequence variants (ASVs) using DADA2 [31]. The latter is a recent method for resolving 16S rRNA gene region variants down to the level of single-nucleotide differences, without imposing the arbitrary dissimilarity thresholds that define operational taxonomic units [32]. Taxonomic assignment was carried out by using the VSEARCH algorithm [33] and the Greengenes database (May 2013 release). Chimeras were discarded during the analysis. Alpha-diversity was evaluated using two different metrics: Faith’s Phylogenetic Diversity (PD whole tree) and observed ASVs. Weighted and Unweighted UniFrac distances were used to construct Principal Coordinates Analysis (PCoA) graphs. All statistical analyses were performed using the R software [34]. PCoA was generated using the “vegan” [35] and “Made4” [36] packages and data separation was tested by permutation test with pseudo-F ratios (function “Adonis” in “vegan”). To compare differences in microbiota composition among groups and covariates at all taxonomic levels and for alpha diversity metrics a preliminary analysis with Wilcoxon rank-sum test or Kruskal–Wallis test was carried out. When necessary, p values were corrected for multiple comparisons using the Benjamini–Hochberg method. A false discovery rate (FDR) ≤ 0.05 was considered as statistically significant. * *p* value ≤ 0.05; ** *p* value ≤ 0.01.

## 3. Results

The 16S rRNA gene sequencing and SCFA determination were performed in a total of 104 and 99 samples, respectively, for twenty pediatric patients sampled before, during and after HSCT. Patients were divided into two groups of ten subjects depending on the type of feeding procedure received right after the transplant, EN or PN. Clinical and transplant characteristics are summarized in Supplementary Table S2. Samples were grouped as “pre” (i.e., samples taken before HSCT), “HSCT” (i.e., the first samples taken after HSCT for all subjects, ranging from 8 to 30 days post-transplant), and “post” (i.e., all other samples taken after HSCT). In order to better visualize the trajectory of GM recovery, post-HSCT samples were further divided into three groups: “post40” (i.e., all samples taken from 31 to 40 days post-HSCT), “post60” (i.e., samples taken from 41 to 60 days post-HSCT) and “post120” (i.e., samples taken from 61 to 120 days after the transplant) (Figure 1).

### 3.1. Variation in the Overall Bacterial Biodiversity in HSCT Patients during Enteral and Parenteral Feeding

**Figure 2:**
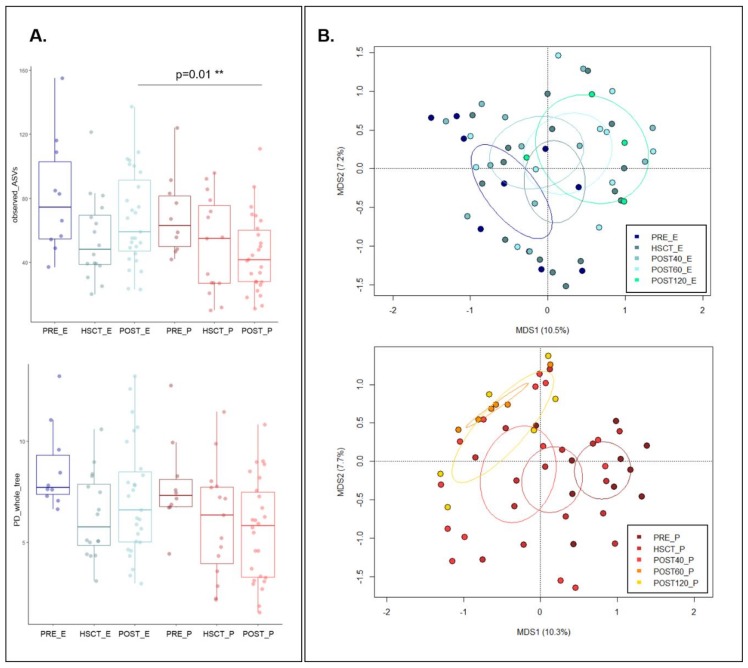
Diversity of pre- and post-HSCT samples in patients treated with enteral and parenteral nutritional regimens. (**A**) Alpha diversity estimated with observed amplicon sequence variants (ASVs, top) and Faith’s Phylogenetic Diversity (PD_whole_tree, bottom) metrics for samples taken at the baseline (PRE), during the transplant (HSCT) and up to 120 days post-HSCT (POST) from patients treated with enteral (E) and parenteral (P) nutrition. Subjects treated with PN (in red) showed a significantly higher number of ASVs (Wilcoxon rank-sum test; *p* = 0.01). (**B**) Principal Coordinates Analysis (PCoA) based on unweighted UniFrac distances between samples taken before (PRE) and after the transplant (HSCT, up to 30 days post-HSCT; POST40, up to 40 days post-HSCT; POST60, from 41 to 60 days post-HSCT; POST120, up to 120 days post-transplant) from patients treated with EN (top) and PN (bottom). A significant separation among groups was observed only in patients treated with PN (permutation test with pseudo-F ratios (Adonis), *p* = 0.001).

The first analysis we carried out was conducted to compare the variation of GM biodiversity from pre- to post-HSCT samples between EN and PN subjects. Different metrics were used to estimate alpha diversity, including phylogenetic diversity and observed ASVs. Both measures indicated, for EN and PN patients, a reduction in GM diversity from pre-HSCT to HSCT samples. However, while PN patients maintained a low level of GM diversity also in post-HSCT samples (Wilcoxon rank-sum test; PRE_P vs. POST_P; observed_asvs: *p* = 0.05; PD_whole_tree: *p* = 0.13), patients fed with EN showed the post-HSCT recovery of a level of GM diversity comparable to that observed in pre-HSCT samples (PRE_E vs. POST_E; *p* = 0.44; *p* = 0.25). These data highlight a significantly different trajectory of GM diversity between EN and PN patients during the course of post-HSCT recovery (POST_E vs. POST_P; *p* = 0.01; *p* = 0.23) (Figure 2A).

In order to explore the variation in the overall GM compositional structure from pre- to post-HSCT in EN and PN patients, a Principal Coordinates Analysis (PCoA) of unweighted UniFrac distances of the GM profiles in the whole sample set was performed. Our data revealed a different GM response to the HSCT perturbation between PN and EN patients. In particular, while the post-HSCT samples from the first showed a significant shift in their GM structure (permutation test with pseudo-F ratios (Adonis); *p* = 0.001) that gradually moved away from the pre-HSCT composition to the one observed up to 120 days post-HSCT (Figure 2B), for EN, the overall GM layout did not undergo a significant dysbiotic shift following HSCT (*p* = 0.063).

### 3.2. Gut Microbiota Composition in Enteral and Parenteral Nutritional Regimen during the HSCT Recovery

**Figure 3:**
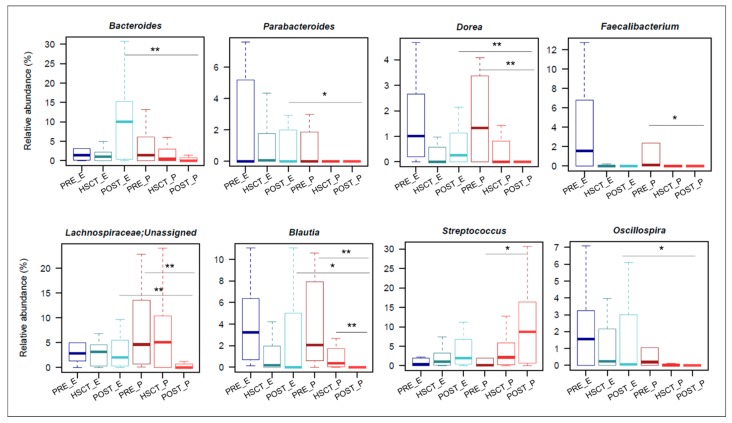
Differences in the gut microbiota composition between HSCT patients following enteral and parenteral nutrition. Boxplots representing the relative abundance distribution of genera that where significantly different between pre-HSCT, HSCT and post-HSCT time points in PN (in red) and EN patients (in blue). * *p* value ≤ 0.05; ** *p* value ≤ 0.01; Wilcoxon rank-sum test.

The GM composition analysis conducted at both the phylum and family level did not highlight any significant difference between EN and PN groups at the baseline (PRE_E vs. PRE_P), as well as the HSCT time points (HSCT_E vs. HSCT_P). In agreement with what has been previously observed by Biagi et al. [4], both for EN and PN patients, HSCT samples were characterized by profound rearrangements of the GM structure, as a result of the HSCT stress (Supplementary Figure S1). Conversely, in the post-HSCT time points, EN and PN patients showed significant differences in the GM compositional structure. Specifically, while in PN subjects pre- and post-HSCT samples showed significant variations in core GM families and genera, in EN patients, the GM composition pre- and post-HSCT did not show any significant divergence (Figure 3).

In particular, in EN patients, post-HSCT, the relative abundance of *Lachnospiraceae* remained stable over time, while it decreased in subjects treated with PN (POST_E vs. POST_P: *p* = 0.003). This difference was accounted for by increased representation of *Blautia* (POST_E vs. POST_P: *p* = 0.036) and *Dorea* (*p* = 0.008), both belonging to *Lachnospiraceae* family, in EN-fed subjects compared to PN patients (Figure 3). A similar increase post-HSCT in EN- vs. PN-fed patients was observed in other genera, such as *Parabacteroides* (*p* = 0.03) and *Oscillospira* (*p* = 0.03). Interestingly, *Faecalibacterium* significantly decreased in post-HSCT samples, compared to the baseline, only in PN patients (Figure 3). Finally, patients fed with EN were characterized by a post-HSCT increase in *Bacteroidaceae* compared to PN patients (POST_E vs. POST_P: *p* = 0.009), mostly attributable to the genus *Bacteroides* (*p* = 0.008). A significant difference was also highlighted for *Streptococcus*, whose relative abundance increased post-transplant in patients fed with PN (PRE_P vs. POST-P: *p* = 0.03), while no difference was observed in EN-fed subjects. Blood culture tests were performed on patients with febrile neutropenia. For EN patients, none of them exhibited evidence of bloodstream infection (BSI). On the other hand, seven PN-treated patients developed eleven documented BSI, mainly attributed to *Staphylococcus* (36.4%), *Enterococcus* (18.2%) and *Streptococcus* (18.2%) genera and mostly identified from 5 to 30 days post-HSCT (Supplementary Table S2).

### 3.3. Short-Chain Fatty Acid Production in EN and PN Patients Undergoing HSCT

**Figure 4:**
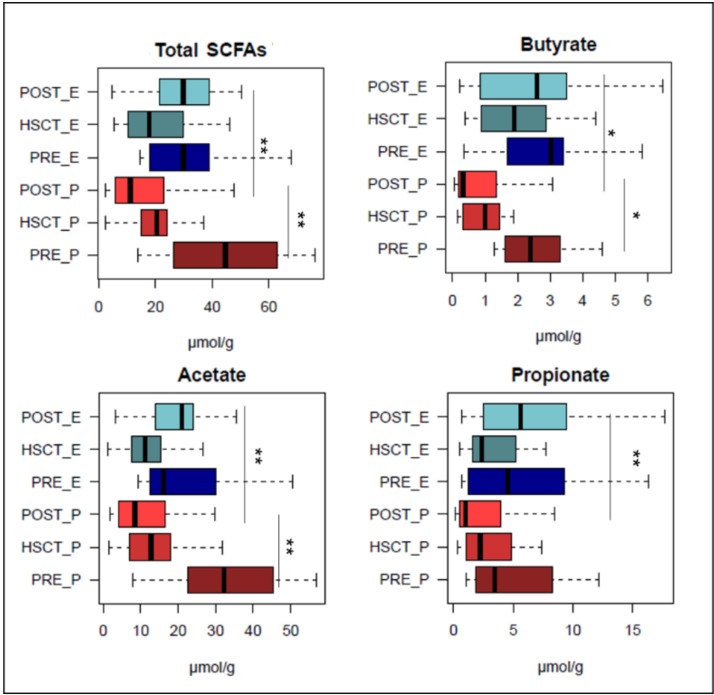
Fecal levels of short-chain fatty acids in HSCT patients after enteral and parenteral nutritional regimens. Boxplots showing the absolute amount distribution for short-chain fatty acids (SCFAs) measured in μmol/g. * *p* value ≤ 0.05; ** *p* value ≤ 0.01; Wilcoxon rank-sum test.

We conducted a gas chromatograph-mass spectrometry (GC–MS) analysis of fecal SCFAs in HSCT patients fed with EN and PN from the baseline (pre-HSCT) to the transplant itself (HSCT samples), up to 120 days after transplant (post-HSCT). According to our findings, patients who received PN were characterized by a significant decrease in SCFA levels in post-HSCT time points compared to pre-HSCT samples (absolute amount (μmol/g) ± SEM, PRE_P vs. POST_P; 44.0 ± 6.2 vs. 16.2 ± 2.9; *p* = 0.006), while for EN subjects, the post-HSCT SCFA production levels were comparable to the baseline (PRE_E vs. POST_E, 35.0 ± 7.4 vs. 31.9 ± 3.1; *p* = 0.78) (Figure 4).

In detail, in post-HSCT samples, EN patients were significantly enriched in butyrate (POST_E vs. POST_P: *p* = 0.01), acetate (*p* = 0.005) and propionate (*p* = 0.005) compared to subjects that received PN. Indeed, a significant decrease in all the major SCFAs, i.e., butyrate (PRE_P vs. POST_P: *p* = 0.01), acetate (*p* = 0.009) and propionate (*p* = 0.08), was observed between pre- and post-HSCT samples of PN patients (Figure 4).

## 4. Discussion

Although post-HSCT enteral feeding is being increasingly recommended [37,38,39], intravenous nutrient intake (PN) is still the first-line nutritional approach for the patients who received HSCT due to its compliance, but it has been associated with several clinical and microbiological adverse effects, including infections and GM dysbiosis [18,19,20,21,22,23,24]. So far, the central role of the GM composition and biodiversity in patients undergoing HSCT has been largely evaluated in several publications, highlighting the disruption of the gut microbial mutualistic asset after the transplant (i.e., until 30 days post-HSCT) compared to the baseline, due to all HSCT conventional treatments that can alter the GM recovery possibilities post-HSCT [4,5,7]. Therefore, in this scenario, we explored the potential of EN in supporting a eubiotic GM trajectory following HSCT. To this end, the GM structure of twenty pediatric patients undergoing HSCT (ten treated with EN and ten with PN) was analyzed at the baseline, right after the transplant and in the post-HSCT recovery. In parallel, we evaluated the GM functionality as SCFAs production by using GC–MS. As shown in several publications [4,5,6,7,40], right after the transplant we observed the loss of the eubiotic GM layout. However, in EN patients, we assisted in the prompt recovery of a diverse eubiotic GM layout, minimizing the dysbiotic shifts following the HSCT. Interestingly, Wilmanski et al. [41] recently found a connection between the eubiotic GM structure and the host blood metabolic profile. This suggests a possible feedback of the eubiotic GM trajectory in HSCT patients also in terms of the blood metabolome, which has been connected with the GvHD risk [42]. At the compositional level, EN-fed subjects recovered a GM structural layout comparable to the one observed before the transplant starting from 30 days post-HSCT, while in PN patients this end point was never achieved. Particularly, we identified some genera including *Faecalibacterum, Dorea*, *Blautia, Bacteroides*, *Parabacteroides* and *Oscillospira*, the relative abundance of which was restored in EN patients during the post-HSCT recovery. Similar findings have very recently been observed in an adult cohort of 23 patients undergoing HSCT by Andersen et al. [43]. Interestingly, most of the microorganisms for which we observed the restoring in EN-fed subjects are well-known health-promoting GM bacteria capable of producing SCFAs [12,44,45,46,47,48,49]. Consistently, the fecal levels of SCFAs, i.e., propionate, butyrate and acetate, were restored only in EN patients between 30 and 120 days post-HSCT. These findings are in line with a recent work showing increased levels of fatty acids in the blood of adult patients receiving mainly EN support post-HSCT [50]. As a matter of fact, intestinal decrease in SCFA levels, especially butyrate, with a loss of GM diversity and SCFA-producers, have been associated with post-HSCT immunological complications [14]. Recently, restoring the physiological levels of endogenous butyrate in in vitro and in vivo models by local administration has been shown to improve intestinal junctional integrity, decrease cellular apoptosis and mitigate GvHD severity [14]. These observations were also demonstrated in an adult cohort that received HSCT, where the increase in the relative abundance of the SCFA-producer *Blautia* was associated with the reduction in GvHD lethality and the improvement of overall patient survival [51]. In this scenario, the potential of EN in keeping a eubiotic and SCFA-producing GM layout may have important post-HSCT clinical implications, as highlighted by the decrease in patient aGvHD severity and the reduction in local and systemic infections in EN compared to PN patients [unpublished observations]. Indeed, for EN subjects, we observed no evidence of BSI, probably supporting less impairment of the intestinal epithelial barrier. On the other hand, PN patients were found have blood culture positivity to several microorganisms (e.g., *Staphylococcus*, *Enterococcus* and *Streptococcus* genera), mainly from day 5 to 30 post-HSCT, leading to systemic infections and clinical complications. Our results regarding the clinical outcomes of EN patients were generally in line with most of the previous studies in literature conducted on adult cohorts post-HSCT [18,19,20,21,22,23]. However, we cannot fail to mention that other studies provide conflicting results for what concern the clinical outcomes of EN assumption in HSCT adult patients [52,53]. In conclusion, in EN patients, we observed prompt GM structural and functional recovery already starting from 30 days post-HSCT, featured by the restoration of health-promoting and SCFA-producing microorganisms and the reduction in full-blown infections. Our data indicate that post-HSCT EN promotes the achievement of GM homeostasis, also including the production of immunomodulating metabolites, in a window frame that is strategic to educate the process of immunological reconstruction, possibly reducing the risk of local and systemic infections and aGvHD onset.

## Supplementary Materials

The following are available online at https://www.mdpi.com/2072-6643/11/12/2958/s1. Figure S1: Gut microbiome composition of pre- and post-HSCT samples of patients fed with enteral and parenteral nutrition. Bar plots indicate the relative abundance of the most represented phyla (A.) and families (B.) in both enteral (E) and parenteral (P) nutrition-fed patients pre-HSCT (PRE), during the transplant (HSCT) and up to 120 days post-HSCT (POST). Only taxa with relative abundance >0.1% in at least two samples were included. Table S1: Nutritional information of enteral diet. Table S2: Anagraphical and clinical information of the twenty enrolled patients.
