# Supplementary material for: Enteral Nutrition in Pediatric Patients Undergoing Hematopoietic SCT Promotes the Recovery of Gut Microbiome Homeostasis
Source: Nutrients. 2019 Dec 4;11(12):2958. doi: 10.3390/nu11122958 (PMC6950621; doi:10.3390/nu11122958)
Supplement: Supplementary file 1 [file nutrients-11-02958-s001.pdf]

Supplementary Figure 1: Supplementary Figure 1: Gut microbiome composition of pre- and post-HSCT samples of patients fed with enteral and parenteral nutrition. Bar plots indicating the relative abundance of the most represented phyla (A.) and families (B.) in both enteral (E) and parenteral (P) feeding patients pre (PRE), during the transplant (HSCT) and up to 120 days post HSCT (POST). Only taxa with relative abundance >0.1% in at least 2 samples were included.

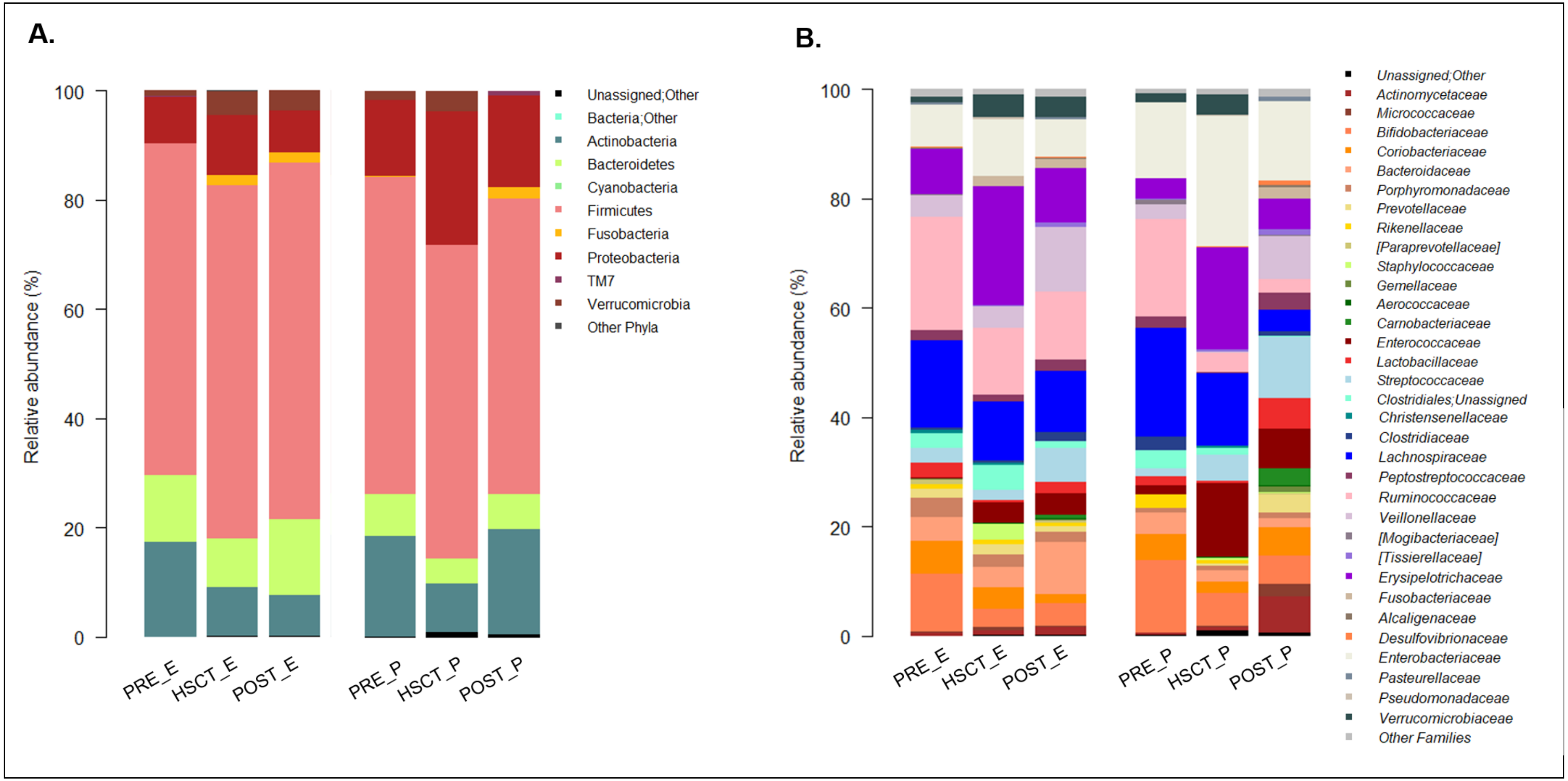

Supplementary Table 1: Nutritional information of enteral diet.

Nutritional Informations for 100 g of product:	
Energy	1992 kJ<br>475 kcal
Fat (42 En%):	22 g
Saturated	9.4 g
Monosaturated	7.7 g
Polysaturated	4.0 g
Carbohydrates (47 En%):	56 g
Sugars	5.0 g
Dietary Fibre (0 En%)	0 g
Proteins (11 En%)	13.3 g
Salt	0.71 g
Vitamins	
Vit.A	214 µg
Vit.D	6.2 µg
Vit.E	6.7 mg
Vit.K	19 mg
Thiamine	0.48 mg
Riboflavin	0.95 mg
Niacin	5.3 mg
Pantothenic acid	1.9 mg
Vit.B_6	0.48 mg
Folate	71.3 µg
Vit.B_{12}	1.2 µg
Biotin	14.3 µg
Vit.C	47.5 mg
Minerals	
Na	285 mg
K	546 mg

Cl	437 mg
Ca	428 mg
P	309 mg
Mg	66.5 mg
Fe	5.7 mg
Zn	5.2 mg
Cu	0.48 mg
Mn	0.29 mg
Mo	24.4 µg
Se	15.2 µg
Cr	7.6 µg
I	71.4 µg
Others	
Choline	95.0 mg
Inositol	26.1 mg
L-carnitine	15.8 mg
Taurine	31.5 mg

Supplementary Table 2: Anagraphical and clinical information of the twenty enrolled patients.

Subject	*Nutritional Regimen*	*Sex/Age(yr)*	*Diagnosis*	*Donor Type*	*Stem cell source*	*Conditioning regimen*	*Engraftment (day)*		*aGvHD*			*Mucositis*	*BSI*	*Outcome at +100*
							PMN (day)	*PLT > 20000 (day)*	*Site*	*Day*	*Corticosteroid resistant*	*Grade*		
E1	EN (+0/+18)	F/8	ALL B	Haploidentical	BM	BU, TT, FLUDARA	+14	+87	Skin ++, Gut ++, LIver +++	+24	n	III		a
E2	EN (+0/+12)	M/18	TM	MMUD	PBSC	TREO,TT,FLUDARA	+14	+20	Skin ++, Gut +	+14	n	/		a
E3	EN (+1/+21)	M/8	ALL B	MMFD	BM	BU, TT, FLUDARA	+16	+33				II		a
E4	EN (+1/+19)	F/8	ALL T	MUD	BM	BU, TT, EDX	+12	+46				III		a
E5	EN (+1/+18)	M/16	AML	Haploidentical	BM	BU, TT, FLUDARA	+23	+41						a
E6	EN (-2/+25)	F/16	AML	Haploidentical	BM	BU, TT, FLUDARA	+22	+35	Skin +, Gut ++	+36	n	II		a
E7	EN (+0/+18)	F/2	HLH	MUD	BM	BU, TT, FLUDARA	+14	+27				II		a
E8	EN (+1/+27)	F/12	RCC	MUD	BM	TREO,TT,FLUDARA	+24	+33				I		a
E9	EN (+0/+22)	F/3	ALL B	MUD	BM	BU, TT, FLUDARA	+19	+19	Skin +, Liver ++	+18	n	I		a
E10	EN (+1/+10); PN (+10/+13)	M/13	ALL B	MUD	BM	BU, TT, FLUDARA	+13	Not Obtained	Skin ++, Gut +	+60	n	II		a

Subject	*Nutritional Regimen*	*Sex/Age(yr)*	*Diagnosis*	*Donor Type*	*Stem cell source*	*Conditioning regimen*	*Engraftment (day)*		*aGvHD*			*Mucositis*	*BSI*	*Outcome at +100*
							PMN (day)	*PLT > 20000 (day)*	*Site*	*Day*	*Corticosteroid resistant*	*Grade*		

P1	PN (+5/+25)	M/15	CGD	MUD	BM	TREO,TT,FLUDARA	+19	+12				II	*S.epidermidis* (+5)	a
P2	PN (+6/+19)	M/7	AML	Haploidentical	BM	BU, TT, FLUDARA	+15	+28				II	*S.aureus* (+7)	a
P3	PN (+0/+16)	M/18	ALL B	MUD	BM	TREO, TT, EDX	+12	+16	Skin ++, Gut +	+28	n	I		a
P4	PN (+0/+18)	F/10	AML	MFD	BM	TREO,TT,FLUDARA	+11	+7				II	*S.epidermidis* (+6); *E.faecalis* (+6)	a
P5	PN (+1/+36)	M/4	JMML	MUD	BM	BU, EDX, L-PAM	+29	+37	Gut ++++	+29	y	I	*E.faecalis* (+57); *S.mitis* (+57); *S.oralis* (+57)	a
P6	EN (+1/+4); PN (+5/+23)	F/1	ALL B	MMUD	BM	BU, TT, EDX	+15	+25				I	*E.coli* (+2)	d
P7	PN (+5/+31)	M/17	RCC	MUD	BM	EDX, FLUDARA, TBI	+28	+36				II	*K.pneumoniae* (+6); *P.aeruginosa* (+15)	a
P8	PN (+4/+13)	M/16	AML	MFD	BM	BU, EDX, L-PAM	+13	+14	Gut +++, Liver +	+21	y	II	*S.aureus* (+94)	a
P9	EN (+2/+4); PN (+5/+20)	M/17	MDS	MUD	PBSC	BU, EDX, L-PAM	+19	+21	Skin ++	+20	n	II		a
P10	PN (+0/+17)	M/18	ALL B	MUD	BM	BU, TT, EDX	+13	+23	Skin +++	+15	n	III		a

Abbreviations: a= alive; aGvHD= acute Graft-versus-Host disease; ALL= Acute Lymphoblastic Leukemia; AML= Acute Myeloid Leukemia; BM= Bone Marrow; BSI= Blood Stream Infections; BU= Busulphan; CGD= Chronic Granulomatous Disease; d= deceased; EDX= Cyclophosphamide; EN = Enteral Nutrition; F=Female; FLUDARA= Fludarabine; HLH= Hemophagocitic LymphoHistiocytosis; JMML= Juvenile Myelomonocytic Leukemia; L-PAM= Melphalan; M= Male; MDS= Myelodisplastic Syndrome; MFD= Matched Familiar Donor; MMFD= Mismatched Familiar Donor; MMUD= Mismatched Unrelated Donor; MUD= Match Unrelated Donor; n= no; PBSC= Peripheral Blood Stem Cell; PLT=Platelets; PMN=Polymorphonuclear Neutrophil; PN= Parenteral Nutrition; RCC= Refractory Citopenia of Childhood; TBI= Total Body Irradiation; TM= Thalassemia Major; TREO= Treosulphan; TT= Thiotepa; y= yes; The plus near by the aGvHD site indicate the grade of severity (from 1 to 4); The numbers between parenthesis indicate the starting and the ending day for the nutritional treatment post-transplant.
